# Human Papillomavirus (HPV) Vaccination Knowledge, Beliefs, and Hesitancy Associated with Stages of Parental Readiness for Adolescent HPV Vaccination: Implications for HPV Vaccination Promotion

**DOI:** 10.3390/tropicalmed8050251

**Published:** 2023-04-26

**Authors:** Seok Won Jin, Yeonggeul Lee, Heather M. Brandt

**Affiliations:** 1 School of Social Work, The University of Memphis, 119 McCord Hall, Memphis, TN 38152, USA; 2 Department of Medical Humanities and Social Science, College of Medicine, Yonsei University, 50-1 Yonsei-ro, Seodaemun-gu, Seoul 03722, Republic of Korea; 3 Social Science Research, University of Seoul, 163 Seoulsiripdaero, Dongdaemun-gu, Seoul 02504, Republic of Korea; lynggl@uos.ac.kr; 4 HPV Cancer Prevention Program, St. Jude Children’s Research Hospital, 262 Danny Thomas Place, Memphis, TN 38105, USA; heather.brandt@stjude.org

**Keywords:** human papillomavirus (HPV), HPV vaccine hesitancy, transtheoretical model, health belief model, Memphis metropolitan areas

## Abstract

The vaccination against human papillomavirus (HPV) has shown effectiveness in preventing six different types of cancer. Despite a safe, effective HPV vaccine, vaccination coverage for adolescents remains suboptimal, especially in the Memphis, Tennessee metropolitan area. Parents/Guardians have a substantial influence on adolescent vaccination, but little is known about parental cognitive factors contributing to intent on adolescent HPV vaccination in this region. Thus, this study examined factors associated with stages of parental readiness for adolescent HPV vaccination by applying the transtheoretical model. A cross-sectional, online survey was conducted to collect quantitative data on sociodemographic characteristics; health-related information; HPV vaccination knowledge, beliefs, and hesitancy; and stages of readiness for adolescent HPV vaccination among parents. Convenience sampling was performed to recruit a total of 497 parents of adolescents aged 11–17 years in Shelby and Tipton Counties in Tennessee and DeSoto County in Mississippi. Binary logistic regression analyses showed that greater knowledge of HPV vaccination, greater perceived susceptibility to HPV, and lower levels of HPV vaccination hesitancy, respectively, distinguished higher from lower stages of parental readiness for adolescent HPV vaccination after controlling for other variables. The findings provide implications for developing readiness for stage-specific interventions targeted to effectively influence the parental decision-making process regarding HPV vaccination for adolescents.

## 1. Introduction

Human papillomavirus (HPV) causes almost all cases of cervical cancers and pre-cancers of the cervix, and the majority of five other cancers of the oropharynx, vagina, vulva, penis, and anus for men and women [1]. In the United States (U.S.), HPV is estimated to account for about 79% of new cases of these cancers annually [2]. This also poses a substantial financial burden—approximately $775 million in direct medical costs—on the U.S. healthcare system [3]. To prevent HPV-attributable cancers, a safe, effective, and durable vaccine against HPV was introduced in the U.S. in 2006 [4,5,6,7,8]. Routine HPV vaccination is recommended for adolescents at age 11 or 12 years and can be started at age 9 [9]. In addition, catch-up vaccination is recommended for eligible individuals, those who are yet not fully vaccinated by the age of 26, and for some people aged 27 to 45 years who are not adequately vaccinated and at risk for new HPV infection with shared clinical decision-making [1].

Despite the widespread availability of the vaccine, HPV vaccination coverage among adolescents remains low in the U.S. [10]. A recent National Immunization Survey-Teen (NIS-Teen) survey showed that in 2021, only 61.7% of adolescents aged 13 through 17 years were up to date with HPV vaccination (i.e., received all the recommended doses of the HPV vaccine according to the guideline) [11], lagging far behind the 80% benchmark of the Healthy People 2030 goal [12]. A sub-analysis of the NIS-Teen data by region also indicated that many of the states in the Mid-South and southeastern U.S. had lower rates of vaccination coverage compared to states in other U.S. regions [13], whereas these states have often experienced high rates of HPV prevalence, HPV-associated cancers, and cervical cancer mortality [14]. Particularly, suboptimal rates of up-to-date HPV vaccination in the target age group were observed in the Memphis, Tennessee metropolitan area, and in Mississippi (32.7%), Tennessee (56.5%), and Arkansas (56.8%), respectively, ranking the lowest, 10th lowest, and 13th lowest across the nation, which were all significantly below the national average and goal as well [13].

Existing literature has indicated that low HPV vaccination coverage among adolescents is strongly associated with parental vaccine hesitancy [15,16]. The World Health Organization (WHO) Strategic Advisory Group of Experts (SAGE) Working Group defined vaccine hesitancy as a behavioral phenomenon of delaying or refusing to receive a vaccine despite the availability of vaccine services [17]. Vaccine hesitancy also was characterized by its complexity, context specificity, and variations by time, place, and type of vaccines, falling on a continuum between full acceptance and outright refusal of the vaccine [17,18]. In 2019, the WHO identified vaccine hesitancy as one of the top threats to public health [19]; for the past few years, the COVID-19 pandemic has elevated levels of hesitancy for vaccines [20]. A recent national online panel survey revealed that 23.0% of U.S. parents in 2019 were hesitant about HPV vaccines, and adolescents living with these vaccine-hesitant parents were significantly less likely than those living with non-vaccine-hesitant parents to receive the vaccine or complete the vaccination series [21]. Multiple studies also demonstrated that common reasons for HPV vaccine hesitancy among parents include limited knowledge or understanding of HPV vaccines, concerns about safety and side effects, medical mistrust, fears of side effects of the vaccine, the link to sexual intercourse, costs, social or religious norms, and complex vaccination decisions [22,23,24,25]. Moreover, a prior study on a national trajectory in vaccine hesitancy among parents showed that parental lack of intent to initiate the HPV vaccination series for adolescents increased from 50.4% in 2012 to 64.0% in 2018 [25]. Another study also showed that reasons for parental HPV vaccine hesitancy had changed over time [26]. For example, between 2008 and 2016, the lack of knowledge about the vaccine declined, while vaccine safety concerns rose during the same period [26].

Literature has also indicated that parental beliefs and knowledge of HPV and the vaccine predict adolescent HPV vaccination. A systematic review indicated that the Health Belief Model (HBM) was the most frequently used theory in improving vaccine knowledge and uptake [27]. According to the HBM, vaccine-related health beliefs are antecedents of the vaccination, so people’s engagement in HPV vaccination results from the interactions among the HBM-based constructs, including perceived susceptibility (to HPV), perceived severity (of HPV-associated diseases), perceived benefits (from HPV vaccination), and/or perceived barriers (to the vaccination) along with cue to action (e.g., provider recommendation) and self-efficacy (e.g., making an appointment for the vaccination) [28]. For example, a person is likely to receive the HPV vaccine when the person perceives increased susceptibility to HPV, increased severity of HPV-associated diseases, increased benefits from HPV vaccination, and reduced barriers to the vaccination along with provider recommendation (i.e., cue to action) and increased ability to make an appointment for the vaccination (i.e., self-efficacy). Additionally, within the HBM, HPV vaccine knowledge functions as a structural variable that influences the decision-making process of vaccine uptake through the four HBM constructs [29,30,31].

Multiple cross-sectional studies found that parental health beliefs based on the HBM constructs were associated with HPV vaccine initiation for adolescent girls [25,32]. In addition, a clinical study revealed that HBM-guided HPV vaccination education coupled with telephone reminders was effective in increasing HPV vaccine uptake and completion of the vaccination series among parents of preteen girls [33]. Furthermore, an HPV education intervention study found that perceived susceptibility among young adult women was significantly linked to HPV vaccine uptake, but not perceived severity or perceived benefits [25]. Several empirical studies also supported the positive connection between parental HPV vaccine knowledge and adolescent vaccine uptake [34,35,36]. As research has indicated that individual beliefs about HPV vaccination can change over time, the associations between the HBM constructs and HPV vaccination can vary.

Likewise, the literature has shown that parental cognitive variables, including hesitancy, beliefs, and knowledge pertaining to HPV and its vaccine can play a critical role in decisionmaking for adolescent HPV vaccination [23,36,37,38]. However, little is still known about how these associations vary according to stages of parental intent on vaccinating adolescents against HPV, thus the cognitive variables evolve continuously [39]. A clearer understanding of these relationships can offer important information for designing stage-specific interventions targeted to effectively influence parental decision-making regarding HPV vaccination for adolescents. Thus, this study aimed to examine how the parental cognitive factors (i.e., HPV vaccination hesitancy, beliefs, and knowledge) were associated with stages of parental readiness for adolescent HPV vaccination using the transtheoretical model of health behavior change (TTM) in addition to the HBM as the conceptual frameworks. The TTM is one of the stages-of-change models that conceptualizes the process of intentional behavior change through the following six stages of change: precontemplation, contemplation, preparation, action, maintenance, and termination [40]. The TTM has been widely used to guide developing interventions directed toward HPV vaccine uptake through assessing the readiness of change for a variety of preventive health behaviors, including cancer screening and vaccination [41,42,43]. Taken together, the research questions of this study were:

Research Question 1: What were the characteristics of the cognitive factors (i.e., HPV vaccination knowledge, beliefs, and hesitancy) among parents?

Research Question 2: How did the characteristics of the cognitive factors differ by stages of readiness for adolescent HPV vaccination among parents?

Research Question 3: What were the cognitive factors that distinguished the stages of readiness for adolescent HPV vaccination among parents?

## 2. Materials and Methods

### 2.1. Study Design and Setting

A descriptive study design with a cross-sectional survey was employed to collect quantitative information on adolescent HPV vaccination among parents in the Memphis, Tennessee metropolitan area. The self-administered online survey was used among participants residing in the three counties of the Memphis metropolitan area, including Shelby and Tipton Counties in Tennessee and DeSoto County in Mississippi. These counties were adjacent to each other right across the Mississippi River, while the Shelby and Tipton Counties constituted the west region of Tennessee, with Shelby County being the first most populated county in Tennessee [44]. As previously noted, these are areas of the U.S. in which suboptimal HPV vaccination coverage persists.

### 2.2. Sampling

Convenience sampling was performed to recruit participants through email-based advertisements via local non-profit organizations and personal referrals. Interested participants were screened for eligibility based on the following criteria: a parent/guardian of a child aged 11 to 17 years and a resident in one of the three counties mentioned earlier. Parents of adolescents who were either under 11 years old or over 17 years old were not included because adolescents were the target population of the HPV vaccination in this study and the Advisory Committee on Immunization Practices (ACIP) recommended routine HPV vaccination at age 11 or 12 years and catch-up vaccination at ages 13 through 17 years among adolescents [45].

### 2.3. Data Collection

Data were collected via an online survey platform (i.e., “Qualtrics”) between March and May 2021 [46]. An online survey approach was chosen over a paper-pencil one to minimize social contact during the COVID-19 pandemic. For data collection, the first author had a series of brainstorming sessions with the HPV Cancer Prevention Program at the St. Jude Children’s Research Hospital as a community research partner. Throughout the sessions, potential community organizations/individuals which/who could distribute recruitment advertisements or refer potential participants were identified, and a survey questionnaire was developed building on existing literature [47,48,49]. An advertisement flyer that included a brief description of the study, an electronic link, and a QR code to the online survey was emailed to the identified potential organizations and individuals. Those who were interested in this study accessed the online survey via the link or QR code provided in the flyer. Individuals accessing the survey were first screened for eligibility, and then eligible individuals only were asked to complete an online consent form. Participants who agreed to participate proceeded to the online survey. The selection of measures in the survey was guided by the existing literature [42,50]. A pilot test of the survey questionnaire with two male and three female parents was conducted to seek input and finalize the survey. The first author’s university institutional review board approved this study (IRB#: PRO-FY2021-297).

### 2.4. Variables

#### 2.4.1. Outcome Variable

The outcome variable of this study was the TTM-based five stages of readiness for adolescent HPV vaccination among participants, consisting of precontemplation, contemplation, preparation, action, and maintenance. Given the nature and schedule of HPV vaccination, the last stage, ‘termination’ of the original six TTM stages was dropped in the present study. To measure the outcome variable, ten response items of the HPV Vaccination Stage of Change Scale used by Patel and colleagues were adopted [51]. For example, the precontemplation stage was assigned when a participant chose a single response item, “I do not plan to get my adolescent vaccinated ever.” The contemplation stage was assigned when a participant chose any of the two response items: “I am unsure about my intention to get my adolescent vaccinated” and “I do not plan to get my adolescent vaccinated in the next 6 months”. The preparation stage was assigned when a participant chose any of the following four response items: “I plan to get my adolescent vaccinated (1st shot) in the next 6 months but have not tried to schedule an appointment”, “I plan to get my adolescent vaccinated (1st shot) in the next month but have not tried to schedule an appointment”, “I have made or tried to make an appointment to discuss HPV vaccination with my adolescent’s medical provider”, and “I have made or tried to make an appointment to get my adolescent vaccinated against HPV”. The action stage was assigned when a participant chose any of the two response items: “My adolescent has received at least 1 shot, but I do not have plans for future shots for him/her” and “My adolescent has received at least 1 shot, and is scheduled to receive the next shot in the HPV vaccine series”. Finally, the maintenance stage was assigned when a participant chose a single response item: “My adolescent has received all recommended shots of the HPV vaccine”.

#### 2.4.2. Independent Variable

Independent variables of this study included HPV vaccination knowledge, beliefs, and hesitancy among participants.

HPV vaccination knowledge. To measure knowledge of HPV vaccination, the eight-item HPV Knowledge Scale used by Karki and colleagues was adopted [52]. The HPV Knowledge Scale included statements about HPV vaccine dose, vaccine effectiveness, and recommended age and sex for receipt of HPV vaccination with options of “true” (=1), “false” (=0), and “don’t know” (=0). Examples of the items include: “The HPV vaccine is given as a single shot”, “The HPV vaccine will prevent all causes of HPV-related cancers”, and “The HPV vaccine is recommended only for women and girls”. Only correct responses were computed, with a greater number of correct answers indicating greater knowledge of the HPV vaccine.

HPV vaccination beliefs. Beliefs about HPV vaccination among the participants were assessed with the 12-item HPV Belief Scale used by Karki and colleagues [52]. The HPV Belief Scale constituted sub-scales of the four HBM-based constructs (i.e., two items of perceived susceptibility, three items of perceived severity, three items of perceived benefits, and four items of perceived barriers) with options on a five-point Likert scale, ranging from strongly disagree to strongly agree. Examples of the items include: perceived susceptibility—“I am at risk of contracting HPV” and “I am at risk of getting HPV associated cancer”; perceived severity—“If I have an HPV infection, it will be disruptive to my health”; perceived benefits—“The HPV vaccine will be effective in preventing HPV infection”; and perceived barriers—“I think the HPV vaccine is unsafe”. Cronbach’s alpha tests of reliability for the four constructs showed 0.561 for perceived susceptibility; 0.612 for perceived severity; 0.537 for perceived benefits; and 0.663 for perceived barriers.

HPV vaccination hesitancy. To assess parental hesitancy for HPV vaccination for adolescents, Szilagyi and colleagues’ modified Vaccine Hesitancy Scale was used [48]. This hesitancy scale included nine items of statements with options on a four-point Likert scale ranging from “strongly disagree” (=1) to “strongly agree” (=4), excluding a neutral response. In this study, eight items of statements out of the original nine items were used because of the similarity between an item of the hesitancy scale and the item of the perceived benefits related to the HPV vaccination beliefs “The HPV vaccine will be effective in preventing HPV infection”. Examples of the items include: “The information I receive about the HPV vaccine from my health care provider is reliable and trustworthy”, “Getting the HPV vaccine is important for the health of others in my community”, and “I am concerned about serious side effects of the HPV vaccine”. All the scores of positive statements were reverse coded and the summed scores were used for data analysis. The higher the mean score was, the higher the level of HPV vaccine hesitancy was. The Cronbach’s alpha of the eight-item scale was 0.731.

#### 2.4.3. Control Variable

The control variables for this study comprised sociodemographic characteristics and health-related information. The sociodemographic characteristics included age, gender, race/ethnicity, educational achievement, employment status, annual household income, and religious fidelity. The health-related information included health insurance, having a primary care provider, self-rated health status, and a number of chronic conditions.

### 2.5. Data Analysis

Descriptive statistics and univariate analysis were performed to examine the sociodemographic characteristics and the variables of interest as well as the stages of readiness for adolescent HPV vaccination among the participants. Bivariate analysis was run to assess these characteristics by the stages of readiness. ANOVA with Scheffe’ test was performed for continuous variables, whereas the chi-squared test could not be used for categorical variables due to a significant portion of cells with the observed value ≤ 5 [53]. Finally, binary logistic regression analyses were used to test associations between the independent variables and the stages of readiness among the participants. For the regression analysis, the five stages of readiness were combined into three types of binaries (lower stages vs. higher stages, with the lower stage as reference) as follows: precontemplation vs. contemplation/preparation/action/maintenance; precontemplation/contemplation vs. preparation/action/maintenance; and precontemplation/contemplation/preparation vs. action/maintenance. List-wise deletion was employed for handling missing data. Stata 14.2 was used for data analysis.

## 3. Results

### 3.1. Sample Characteristics

**Figure 1:**
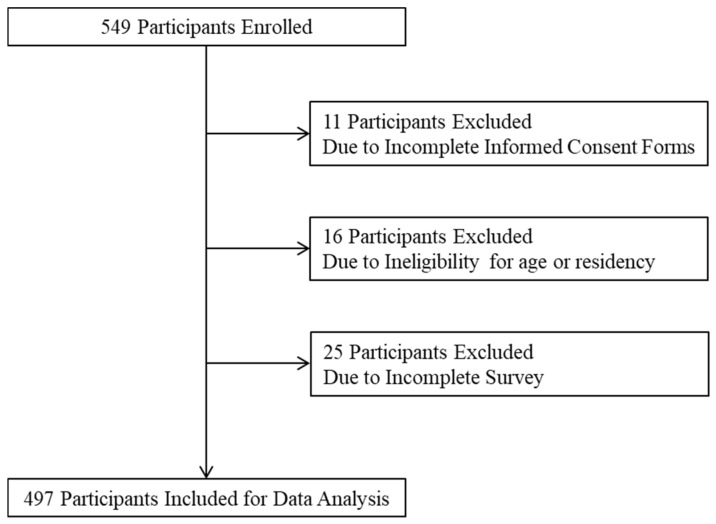
A schematic diagram of the selection of participants for data analysis.

As shown in Figure 1, a total of 549 parents of adolescents were enrolled to participate, and 497 participants (90.5%) who completed the survey were included in the analysis.

Table 1 shows the sociodemographic and health-related characteristics of the participants. More than half (54.7%) were female. The mean age of the participants was 39.6 years (SD = 6.02), with the majority approaching their thirties (40.1%) or forties (53.7%). About 79% were white, and about 12% and 9% were Black and from other races, respectively. In addition, the majority were employed (95.5%) and had an annual household income of $40,000 or above (87.4%). Roughly 93% reported having health insurance, and nearly 80% reported having a primary care provider, while above one-third (35.5%) had at least one chronic condition. Finally, about 94% reported that religion was important. Compared to the characteristics of Memphis, Tennessee-Mississippi-Arkansas Metro Area, the sample was dominantly white (41% vs. 79%) [54]. Table 1 also presents distributions of the stages of readiness for adolescent HPV vaccination among the participants. The descriptive analysis showed that 4.2% were categorized in the precontemplation stage; 23.1% in the contemplation stage; 57.5% in the preparation stage; and 14.7% in the action or maintenance stage.

### 3.2. Independent Variables by Stages of Readiness for Adolescent HPV Vaccination

Table 2 shows the results of descriptive analysis and ANOVA with Scheffe test on the cognitive variables as follows: HPV vaccination knowledge, HBM-based beliefs of HPV vaccination (i.e., perceived susceptibility, perceived severity, perceived benefit, and perceived barrier), and HPV vaccination hesitancy. The mean score of HPV vaccination knowledge was 4.5 (SD = 2.50) with a possible score range from 0 to 8. There was a significant mean difference (*F* = 11.95, *p* < 0.001) in HPV vaccination knowledge between the stages of contemplation (M = 3.4, SD = 2.30) and preparation (M = 5.0, SD = 2.45). For the mean scores of the four HBM-based constructs, perceived susceptibility was 5.8 (SD = 1.27) with a possible score range from 2 to 8; perceived severity was 9.0 (SD = 1.81) with a possible score range from 3 to 12; perceived benefit was 9.0 (SD = 1.68) with a possible score range from 3 to 12; and perceived barrier was 10.8 (SD = 2.36) with a possible score range from 4 to 16. There were significant mean differences in perceived susceptibility (*F* = 6.10, *p* < 0.001), perceived severity (*F* = 3.47, *p* < 0.05), and perceived benefit (*F* = 7.58, *p* < 0.001), respectively, between at least one pair of the stages of readiness; there was no significant mean difference in perceived barrier between the stages of readiness. Finally, the mean score of HPV vaccination hesitancy was 17.5 (SD = 3.05) with a possible score range from 8 to 32. Participants with lower scores of HPV vaccine hesitancy tended to be in higher stages of readiness for adolescent HPV vaccination (*F* = 7.72, *p* < 0.001).

### 3.3. Factors Associated with Stages of Readiness for Adolescent HPV Vaccination

Binary logistic regression analyses were performed to examine the associations of the independent variables with stages of readiness for adolescent HPV vaccination among the participants. Table 3 shows the results.

#### 3.3.1. Precontemplation Stage vs. Contemplation/Preparation/Action/Maintenance Stage

The results showed that no variables of interest were associated with the higher stage of readiness (contemplation/preparation/action/maintenance) compared to the lower stage (precontemplation), controlling for other variables. Among the control variables, however, age (Odds Ratio [OR] = 1.26, 95% Confidence Intervals [CI: 1.12, 1.42]) and annual household income (OR = 1.79, 95% CI [1.04, 3.07]) were significantly associated with the higher stage of readiness compared to the lower one.

#### 3.3.2. Precontemplation/Contemplation Stage vs. Preparation/Action/Maintenance Stage

The independent variables of interest that distinguish parents in the stage of precontemplation/contemplation from those in the stage of preparation/action/maintenance were investigated. The regression analysis showed that parents who had reported greater HPV vaccination knowledge (OR = 1.28, 95% CI [1.15, 1.43]) and greater perceived susceptibility (OR = 1.36, 95% CI [1.10, 1.69]), respectively, were likely to be in the higher stage of readiness for adolescent HPV vaccination (preparation/action/maintenance) compared to the lower one (precontemplation/contemplation), controlling for other variables. For the control variables, the analysis showed that parents who were male (OR = 0.61, 95% CI [0.38, 0.98]) and had reported two or more chronic conditions (OR = 0.41, 95% CI [0.21, 0.80]), respectively, were less likely to be in the higher stage of readiness compared to the lower one.

#### 3.3.3. Precontemplation/Contemplation/Preparation Stage vs. Action/Maintenance Stage

The independent variables of interest that distinguish parents in the stage of precontemplation/contemplation/preparation from those in the stage of action/maintenance were investigated. The analysis demonstrated that parents who had reported lower scores of HPV vaccination hesitancy (OR = 0.86, 95% CI [0.77, 0.98]) were significantly associated with a higher stage of readiness for adolescent HPV vaccination compared to the lower one, controlling for other variables. In this analysis, parents who had self-identified as white (OR = 0.32, 95% CI [0.13, 0.79]) or other races (OR = 0.05, 95% CI [0.01, 0.25]) were less likely to be in the higher stage of readiness compared to the lower one. In comparison, parents who had reported higher annual household income (OR = 1.54, 95% CI [1.17, 2.01]) were more likely to be in the higher stage of readiness compared to the lower one. Finally, parents who had a primary healthcare provider (OR = 0.35, 95% CI [0.18, 0.71]) were less likely to be in the higher stage of readiness for adolescent HPV vaccination compared to the lower one.

## 4. Discussion

Vaccination hesitancy occurs on a continuum that requires additional exploration to inform appropriately aligned intervention strategies in order to improve HPV vaccination [21,24,55,56,57]. HPV vaccination knowledge and beliefs form the foundation of theoretical underpinnings to describe relationships with HPV vaccination. In this study, TTM [58] and HBM [59] were operationalized through construct-guided measurement to understand how a level or stage of readiness aligned with independent variables, including HPV vaccination knowledge, beliefs, and hesitancy. Similar to previously published research [60,61,62,63,64,65,66,67], primarily in college students and young adults, results showed identified relationships between the stage of readiness and HPV vaccination knowledge, beliefs, and hesitancy. The majority of respondents in the current study were in the preparation stage with fewer in precontemplation, contemplation, and action or maintenance stages. Higher levels of HPV vaccination knowledge and lower HPV vaccination hesitancy resulted in a higher stage of readiness. In addition, the age of the parent (older), annual household income (higher), and having a primary healthcare provider were also associated with a higher stage of readiness. These independent predictors of a higher stage of readiness in this study have been previously shown to predict higher levels of HPV vaccination coverage. In this regard, the contribution of this study is not elucidating new predictors or correlations but rather emphasizing the TTM and HBM measurement approach to better understand determinants and inform intervention activities.

Therefore, there are several implications for interventions based on the results. The theoretically-informed measurement approach derived from TTM and HBM focused on identifying stages of readiness and alignment with various independent variables. One practical way in which these results have implications for interventions is the operationalizing of results into intervention activities. For example, the results indicate parents/guardians who possessed lower levels of HPV vaccination hesitancy were more likely to be in the preparation stage. Future interventions should focus on the activation of those in the preparation stage who have demonstrated generally high levels of belief and trust in HPV vaccination and a willingness to act on this information. According to TTM [50,58,68], motivational strategies to strengthen parents’/caregivers’ commitment, e.g., offering incentives, enhancing social support, using persuasive communication, and addressing barriers, can mobilize them to make a firm decision to act, i.e., vaccinate their children. In this study, respondents had high levels of healthcare access and routine provider care, so facilitating connections to HPV vaccination access would be an ideal aspect of the intervention. It is critical to utilize motivational strategies that mobilize parents’ decision to vaccinate their children. Upon the availability of HPV vaccination, the assessment of the decisional balance (pros vs. cons) of getting vaccinated becomes more favorable and presents an opportunity to make an informed decision. Rather than focusing on increasing awareness and knowledge, addressing negatively held beliefs, and working to build confidence and reduce hesitancy, the proportion in preparation was ready to act on this information. Particularly, building vaccine confidence requires a multifaceted approach that enhances provider communication (strong provider recommendation using presumptive language) [69,70,71,72,73], addresses concerns over side effects [74], and uses peer influence [75,76]. By employing these strategies, interventions can increase vaccine confidence and ultimately increase vaccination. Future intervention research could include an assessment of the stage of readiness and employ appropriately aligned activation strategies.

In addition to conveying the value of understanding the stage of readiness and associated determinants, this study attempted to identify differentiation between precontemplation/contemplation stages (i.e., consideration stages) and preparation/action/maintenance stages (i.e., action stages). This analytical grouping of theory-guided stages may have resulted in the misclassification of respondents [75]. The Patel et al. scale has been previously validated using single items to assess the stage of readiness [51]. The misclassification of respondents could have occurred among respondents for whom items did not attend to decisional balance between or across the stage of readiness. It is possible for people to vacillate from one stage to the next or regress to a previous stage based on new information and experiences [76]. This is common in decisional balance wherein new information is constantly being considered in comparison to what was known previously. Future studies may want to expand understanding of the stages to more accurately understand the stage of readiness over time.

### Limitations

The present study offered insight into drivers of HPV vaccination coverage in a population with historically low coverage. No new relationships correlated with predicting HPV vaccination were discovered. However, the value of understanding the stage of readiness has important implications for intervention strategies. While there are several strengths, such as the study population, theoretically-informed measurement approach, and relatively large sample size, limitations also exist. Firstly, this study employed convenience sampling which limits representations of the findings for this group. Future research should consider probability sampling. In addition, this study might involve recall bias because parents were asked to recall the vaccination status of their adolescents. Future studies need to collect information related to adolescent vaccination status using immunization information systems, vaccination cards, or visual aids such as vaccination charts. The composition of the sample, in spite of being relatively large, lacked diversity in race and ethnicity (i.e., predominately white) and health insurance access (i.e., high levels of insurance and regular provider reported). The study was conducted during the COVID-19 pandemic, which also may have influenced interest and participation resulting in a less diverse sample. Other limitations include the cross-sectional nature of the study, which provides only a snapshot of the stage of readiness, which may change over time and may be influenced by a range of factors, especially during the continued duration of the COVID-19 pandemic. The results are informative but must be considered within these and potentially other limitations prior to generalizing other populations.

## 5. Conclusions

This study examined factors associated with stages of parental/guardian readiness for adolescent HPV vaccination, applying the TTM and HBM. The findings provide implications for developing readiness for stage-specific interventions targeted to effectively influence parental decision-making regarding HPV vaccination for adolescents. These efforts are particularly important in areas where there is low HPV vaccination coverage. Not all people who are in these areas may have low awareness and knowledge and/or high levels of hesitancy. In some instances, those parents/caregivers with unvaccinated children may need activation strategies to connect their stages of readiness with appropriate actions in light of the availability and accessibility of HPV vaccinations. Additional theoretically-guided studies of parents/caregivers in areas with low HPV vaccination coverage are needed to inform intervention strategies and assess implemented interventions for effectiveness in increasing HPV vaccination for cancer prevention.

## Figures and Tables

**Table 1:** Analysis of sample characteristics by stages of parental readiness for adolescent HPV vaccination (*n* = 497).

Variables	Total *n* = 495		Precontemplation *n* = 21		Contemplation *n* = 115		Preparation *n* = 286		Action/Maintenance *n* = 73	
	*n*	%	*n*	%	*n*	%	*n*	%	*n*	%
Age (mean = 39.6, SD = 6.02)										
≤29	20	4.31	5	29.41	5	4.55	8	3.00	2	2.86
30–39	186	40.09	11	64.71	44	40.00	96	35.96	35	50.00
40–49	249	53.66	1	5.88	58	52.73	158	59.18	32	45.71
≥50	9	1.94	0	0.00	3	2.73	5	1.87	1	1.43
Gender										
Female	264	54.66	13	68.42	43	39.45	166	58.66	42	58.33
Male	219	45.34	6	31.58	66	60.55	117	41.34	30	41.67
Race										
Black	57	11.66	8	42.11	14	12.17	22	7.80	13	17.81
White	387	79.14	9	47.37	96	83.48	225	79.79	57	78.08
Others	45	9.20	2	10.53	5	4.35	35	12.41	3	4.11
Employment										
Employed	463	95.46	19	100.00	100	89.29	272	96.80	72	98.63
Unemployed	22	4.54	0	0.00	12	10.71	9	3.20	1	1.37
Annual household income										
<20,000	16	3.30	6	31.58	1	0.88	7	2.49	2	2.78
$20,001–$40,000	45	9.28	1	5.26	14	12.39	30	10.68	0	0.00
$40,001–$60,000	197	40.62	6	31.58	46	40.71	123	43.77	22	30.56
$60,001–$80,000	110	22.68	5	26.32	16	14.16	63	22.42	26	36.11
$80,001–$100,000	85	17.53	1	5.26	29	25.66	42	14.95	13	18.06
>$100,000	32	6.60	0	0.00	7	6.19	16	5.69	9	12.50
Insurance										
Yes	454	93.42	18	94.74	105	92.92	262	93.24	69	94.52
No	32	6.58	1	5.26	8	7.08	19	6.76	4	5.48
Primary healthcare provider										
Yes	386	79.42	17	89.47	87	76.99	231	82.21	51	69.86
No	100	20.58	2	10.53	26	23.01	50	17.79	22	30.14
Health status										
Very poor/poor/fair	157	32.3	6	31.58	41	36.28	94	33.46	16	21.92
Good or excellent	329	67.7	13	68.42	72	63.72	187	66.55	57	78.08
Number of chronic conditions										
None	313	64.54	12	66.67	63	55.75	189	67.26	49	67.12
One	100	20.62	1	5.56	21	18.58	65	23.13	13	17.81
Two or more	72	14.85	5	27.78	29	25.66	27	9.61	11	15.07
Religiosity										
Important	451	93.96	15	78.95	106	96.36	262	93.91	68	94.44
Not important	29	6.04	4	21.05	4	3.64	17	6.09	4	5.56

**Table 2:** Analysis of independent variables by stages of parental readiness for adolescent HPV vaccination (*n* = 497).

Variables	Total *n* = 495		Precontemplation (a) *n* = 21		Contemplation (b) *n* = 115		Preparation (c) *n* = 286		Action/Maintenance (d) *n* = 73		F (*p*-Value)	Scheffe
	Mean	SD	Mean	SD	Mean	SD	Mean	SD	Mean	SD		
HPV vaccine knowledge (Range: 0–8)	4.50	2.50	3.71	3.05	3.44	2.30	4.99	2.45	4.45	2.33	11.95 (0.000)	b < c
Health Belief Model												
Susceptibility (Range: 2–8)	5.82	1.27	5.71	1.59	5.49	1.33	6.02	1.20	5.59	1.22	6.10 (0.000)	b < c
Severity (Range: 3–12)	8.98	1.81	8.81	2.02	8.55	1.92	9.17	1.76	8.93	1.55	3.47 (0.016)	b < c
Benefit (Range: 3–12)	8.94	1.69	8.29	2.15	8.39	1.81	9.15	1.55	9.16	1.63	7.29 (0.000)	b < c,d
Barrier (Range: 4–16)	10.84	2.36	10.81	2.48	10.91	2.36	10.98	2.26	10.22	2.62	2.09 (0.101)	-
HPV vaccine hesitancy (Range: 8–32)	17.47	3.05	18.90	4.30	18.28	2.70	17.28	2.92	16.55	3.33	7.06 (0.000)	a > d b > c,d

**Table 3:** Logistic regression analysis of factors associated with stages of parental readiness for adolescent HPV vaccination (*n* = 497).

Variables	Precontemplation vs. Contemplation/Preparation/Action/Maintenance			Precontemplation/Contemplation vs. Preparation/Action/Maintenance			Precontemplation/Contemplation/Preparation vs. Action/Maintenance		
	*OR*	[95% CI]	*p*	*OR*	[95% CI]	*p*	*OR*	[95% CI]	*p*
HPV vaccine knowledge	0.94	[0.72, 1.23]	0.636	1.28	[1.15, 1.43]	0.000	0.98	[0.86, 1.11]	0.735
Health Belief Model									
Susceptibility	1.03	[0.59, 1.79]	0.916	1.36	[1.10, 1.69]	0.004	0.87	[0.68, 1.10]	0.237
Severity	0.84	[0.50, 1.40]	0.500	0.94	[0.81, 1.11]	0.473	0.95	[0.79, 1.14]	0.569
Benefit	1.20	[0.74, 1.95]	0.451	1.19	[0.99, 1.43]	062	1.09	[0.87, 1.35]	0.470
Barrier	1.03	[0.75, 1.42]	0.839	1.00	[0.89, 1.13]	0.950	0.98	[0.86, 1.12]	0.777
HPV vaccine hesitancy	0.95	[0.73, 1.24]	0.718	0.97	[0.87, 1.08]	0.555	0.86	[0.77, 0.98]	0.018
Age	1.26	[1.12, 1.42]	0.000	1.00	[0.96, 1.05]	0.835	0.98	[0.93, 1.03]	0.380
Gender (ref. = female)									
Male	1.57	[0.43, 5.72]	0.496	0.61	[0.38, 0.98]	0.039	1.01	[0.57, 1.79]	0.978
Race (ref. = black)									
White	1.65	[0.35, 7.87]	0.528	0.73	[0.33, 1.61]	0.437	0.32	[0.13, 0.79]	0.013
Others	0.57	[0.07, 5.02]	0.616	1.53	[0.48, 4.83]	0.473	0.05	[0.01, 0.25]	0.000
Employment (ref. = unemployed)									
Employed	1.00	[-,-]	-	2.28	[0.86, 6.04]	0.096	2.87	[0.35, 23.59]	0.327
Annual household income	1.79	[1.04, 3.07]	0.036	1.16	[0.92, 1.45]	0.201	1.54	[1.17, 2.01]	0.002
Insurance (ref. = no)									
Yes	3.31	[0.15, 72.22]	0.447	0.75	[0.27, 2.07]	0.578	1.60	[0.47, 5.50]	0.452
Primary healthcare provider (ref. = no)									
Yes	0.25	[0.03, 2.43]	0.235	0.87	[0.46, 1.62]	0.650	0.35	[0.18, 0.71]	0.004
Health status (ref. = very poor/poor/fair)									
Good or excellent	0.47	[0.11, 1.97]	0.300	0.70	[0.41, 1.18]	0.178	1.39	[0.71, 2.72]	0.331
Number of chronic conditions (ref. = none)									
One	4.91	[0.49, 49.17]	0.176	1.31	[0.69, 2.48]	0.408	0.83	[0.39, 1.79]	0.636
Two or more	0.99	[0.20, 4.97]	0.992	0.41	[0.21, 0.80]	0.009	0.83	[0.34, 2.03]	0.688
Religiosity (ref. = Not important)									
Important	3.90	[0.60, 25.41]	0.154	0.79	[0.27, 2.30]	0.669	1.37	[0.37, 5.11]	0.639
Number of observations	427			448			448		
$\mathrm{LR}\;\chi^{2}$ (*p*)	41.91 (0.001)			72.46 (0.000)			45.40 (0.000)		
$\mathrm{McFadden}’s\;R^{2}$	0.307			0.141			0.119		

## Data Availability

Not applicable.
